# Supplementary material for: Systematic Review: Neurodevelopmental Benefits of Active/Passive School Exposure to Green and/or Blue Spaces in Children and Adolescents
Source: Int J Environ Res Public Health. 2023 Feb 23;20(5):3958. doi: 10.3390/ijerph20053958 (PMC10001910; doi:10.3390/ijerph20053958)
Supplement: Supplementary file 1 [file ijerph-20-03958-s001.zip › Supplementary Material S1.pdf]

Table S1. Search strategy.

Database	Keyword Search
PubMed	(parks, recreational OR blue space OR green space OR natural outdoor) AND (School) AND (neurobehavioral manifestations OR child development OR neurodevelopmental disorders OR neurodevelopment OR Neurobehavior OR Mental health OR academic performance)
Scopus	("green space" OR "blue space") AND school AND ("neurodevelopment" OR "neurobehavioral" OR "academic performance") AND (LIMIT-TO (PUBYEAR , 2022) OR LIMIT-TO (PUBYEAR , 2021) OR LIMIT-TO (PUBYEAR , 2020) OR LIMIT-TO (PUBYEAR , 2019) OR LIMIT-TO (PUBYEAR , 2018) OR LIMIT-TO (PUBYEAR , 2017))
Cochrane	(green space OR blue space) AND (School) AND (neurodevelopment OR neurobehavioral OR academic performance OR mental health)
GreenFILE	Boolean/Phrase:<br>(green spaces or outdoor spaces or natural spaces) AND school AND (Neurodevelopment or neurocognitive)<br>(green spaces or outdoor spaces or natural spaces) AND school AND (neurobehaviour or neurobehavioral development)
Scielo	Todos los índices. (Espacio verde) AND (Colegio) AND (Neurodesarrollo).<br>Todos los índices. (Espacio verde) AND (Colegio) AND (Neurocomportamiento).<br>Todos los índices. (Espacio azul) AND (Colegio) AND (Neurodesarrollo).<br>Todos los índices. (Espacio azul) AND (Colegio) AND (Neurocomportamiento).
