# Supplementary material for: Systematic Review: Neurodevelopmental Benefits of Active/Passive School Exposure to Green and/or Blue Spaces in Children and Adolescents
Source: Int J Environ Res Public Health. 2023 Feb 23;20(5):3958. doi: 10.3390/ijerph20053958 (PMC10001910; doi:10.3390/ijerph20053958)
Supplement: Supplementary file 1 [file ijerph-20-03958-s001.zip › Supplementary Material S2.pdf]

Table S2. QuADS scores breakdown by study.

	Macnaughton et al (2017)	Kweon et al (2017)	Hodson et al (2017)	Scott et al (2018)	Sivarajah et al (2018)	Kuo et al (2018)	Browning et al (2018)	Yang et al (2019)	Markevych et al (2018)	Leung et al. (2019)	Liao , Jiaqiang, et al., (2020)	Bernardo et al., (2021)	Kuo , Ming, et al. (2021)	Ezpeleta et al. (2022)	Almeida et al (2022)	Weeberb and (2022)	Bijnens , et al. (2022)	Wu et al (2017)	Chiumento et al (2018)	Anabitarte et al (2021)	Norwood et al (2021)	Bates et al (2018)	Miygind et al (2018)	Friedman et al (2022)	Carver et al (2022)	Amicone et al (2018)	Julvez et al (2021)	Long-Wight et al(2018)	Mean score
1.Theoretical or conceptual underpinning to the research	3	3	3	3	2	3	3	2	3	3	1	3	3	2	3	3	3	2	2	2	3	3	3	3	2	3	3	2	2.6
2. Statement of research aim/s	1	3	1	3	2	3	2	2	2	3	1	3	3	2	2	1	2	2	1	2	3	3	3	3	2	3	3	2	2.3
3. Clear description of research setting and target population	2	3	3	3	3	3	1	3	3	2	3	3	3	3	3	2	2	3	3	3	3	3	3	3	3	3	3	2	2.8
4.The study design es appropriate to address the stated research aim/s	3	3	3	3	2	3	3	3	3	3	2	3	3	3	3	2	3	2	3	2	3	3	3	2	3	3	3	2	2.8
5.Appropriate sampling to address the research aim/s.	1	2	2	3	3	3	2	3	3	2	3	2	3	3	3	2	2	3	2	2	3	2	3	2	3	2	2	2	2.4
6. Rationale for choice of data collection tool/s	2	3	2	3	2	3	2	3	3	3	3	3	3	3	2	3	3	3	3	2	2	2	3	2	3	2	3	2	2.6
7.The format and content of data collection tools es appropriate to address the stated research aim/s	3	3	3	3	2	3	3	2	3	3	3	3	3	3	3	3	3	3	3	2	2	2	3	2	2	3	3	1	2.7
8. Description of data collection process	2	3	3	3	2	3	1	3	3	2	3	3	3	3	2	2	2	2	2	2	2	2	3	1	3	3	3	2	2.4
9. Recruitment data provided	2	2	3	3	2	3	2	3	3	3	2	2	3	2	2	1	2	2	2	2	3	3	3	2	2	2	3	2	2.4
10.Justification for analytical method selected	2	3	3	3	3	3	3	3	3	3	3	1	3	3	3	2	3	3	2	3	2	2	3	2	3	2	3	3	2.7
11.The method of analysis was appropriate to answer the research aim/s	3	3	3	3	3	3	3	3	3	3	2	2	3	3	3	2	3	3	2	3	2	1	3	1	1	1	3	3	2.5
12. Evidence that the research stakeholders have been considered in research design or conduct.	1	2	2	2	1	3	2	2	2	2	2	1	2	2	1	1	2	2	2	2	1	2	2	2	2	2	2	1	1.8
13. Strengths and limitations critically discussed	1	3	2	3	1	3	2	3	3	3	2	1	3	2	3	3	3	3	2	2	3	2	3	3	2	3	3	2	2.5

**Total score (maximum 39)**	**26**	**36**	**33**	**38**	**28**	**39**	**29**	**35**	**37**	**35**	**30**	**30**	**38**	**3. 4**	**33**	**27**	**33**	**33**	**29**	**29**	**32**	**30**	**38**	**28**	**31**	**32**	**37**	**26**
**Mean score**																												**32.4**
